# The association between physical and sensory function, self-perceived health, and 24-hour activity patterns for older people: A compositional data analysis

**DOI:** 10.1371/journal.pone.0340216

**Published:** 2025-12-31

**Authors:** Dan Li, Mi Zhou, Xiaomei Song

**Affiliations:** 1 Follow-up Office, The Second Affiliated Hospital of Soochow University, Suzhou, Jiangsu, China; 2 Allied Health and Human Performance, University of South Australia, Adelaide, Australia; 3 Department of Nursing, The Second Affiliated Hospital of Soochow University, Suzhou, Jiangsu, China; Universiti Malaya, MALAYSIA

## Abstract

**Background and objectives:**

Older adults’ physical and sensory function and self-perceived health state are associated with their daily health behavior, such as moderate-to-vigorous physical activity (MVPA), light physical activity (LPA), sedentary behavior (SEB), and sleep duration (SD), though most studies examine these independently, overlooking 24-hour interactions. This study aims to investigate the relationships between physical and sensory function (vision, hearing, activity limitations), self-perceived health and the pattern of 24-hour activity behaviors via compositional data analysis.

**Methods:**

A secondary data analysis was conducted on data from the Survey of Health, Ageing and Retirement in Europe. Compositional data analysis was employed to account for the inherently interdependent nature of these behaviors. Linear regression models were implemented, designating activity behaviors as the dependent variable and sensory/physical function as the independent variable.

**Results:**

The results indicated that vision and hearing showed weaker and nonsignificant associations with activity behaviors. Activity limitations significantly influence health behavior patterns, with no limitations associated with more time in MVPA and less time in SEB and SD. Self-perceived health is significantly positively associated with more MVPA, while inversely associated with SEB and SD.

**Conclusion:**

This study revealed that older adults with limitations in their daily activities showed the most sedentary daily activity pattern. Future research should develop targeted interventions to improve activity behaviors in this population.

## 1. Introduction

A deficiency in physical activity (PA), sedentary behavior (SEB), and sleep correlates with increased mortality rates and chronic diseases among older adults [1–3]. Research suggests that encouraging healthier lifestyles for older adults—such as regular PA and reducing SEB—can help prevent chronic diseases and reduce mortality [4]. However, owing to the decline in sensory and physical function, health guidelines and lifestyle recommendations for these patients are sometimes challenging to implement [5,6]. Previous studies indicate that, due to deteriorations in vision and hearing, older adults generally favor staying in safer environments, hence reducing their engagement in activities [7,8]. Furthermore, they encounter substantial difficulties in obtaining and adhering to lifestyle suggestions from healthcare providers [8]. These difficulties frequently stem from physical limitations, as their restricted mobility and diminished functional independence hinder their ability to incorporate suggested behaviors into their routines. Consequently, individuals of this group are less inclined to adopt pertinent recommendations, further exacerbating their health challenges [9,10].

Despite extensive research investigating the relationship between declines in sensory and physical function and lifestyle, many studies consider these activity behaviors in isolation when examining their associations, overlooking the interactions between different behaviors within a 24-hour timeframe [11–14]. Such interactions may cause misleading correlations and collinearity problems in the analysis of time-use data. PA, sleep, and SEB constitute an interdependent pattern of one-day activity behaviors, wherein an increase in one behavior necessarily decreases the time allocated for others. This siloed approach can over- or under-estimate individual activity behavior effects.

In recent years, compositional data analysis (CoDA) has garnered significant attention due to its ability to provide the simultaneous analysis of all components using log-ratio transformations [15,16]. This methodology mitigates prevalent challenges in conventional statistical analysis, and enables researchers to model the relationships between sensory and physical function and time allocation. Although a systematic review of CoDA studies has explored the relationships between 24-hour movement behaviors and adult health outcomes [17], prior work has focused chiefly on indicators such as obesity, cardio-metabolic health markers, and all-cause mortality. To date, no study has employed CoDA to examine time allocation patterns in individuals with varying sensory and physical function levels.

This study aims to differentiate the time allocation across four activity behaviors for individuals with varying eyesight, hearing, daily activity limitations, and self-perceived health, using a CoDA framework. The objective is to analyze the relationships between 24-hour activity behavior patterns and certain sensory/physical function variables.

## 2. Materials and methods

### 2.1. Procedure

We conducted a cross-sectional secondary data analysis by utilizing data from the Survey of Health, Ageing and Retirement in Europe (SHARE), a cross-national, multidisciplinary database consisting of samples of community-based adults aged 50 years or older [18]. The principle of secondary data analysis is to address new research questions by reanalyzing existing datasets, thereby maximizing data utility while minimizing time, cost, and ethical concerns associated with primary data collection. The initial data collection was carried out in 2004, with subsequent data waves collected at biennial intervals. The present study utilized accelerometer and physical health data from wave 8 (collected in 2019/2020), involving 46,733 participants from 27 countries. All participants included were anonymous. The data collection commenced in October 2019 and was suspended in March 2020 due to the outbreak of COVID-19 [19]. Use of the SHARE data (8th wave) was approved by the Ethics Committee of the Max Planck Society for the Progress of Science. SHARE-ERIC’s activities related to human subjects research are guided by international research ethics principles such as the Respect Code of Practice for Socio-Economic Research and the Declaration of Helsinki.

### 2.2. Measures

#### 2.2.1. 24h movement behavior.

Accelerometer data were collected from a subsample in ten Europen countries: Belgium, Czech Republic, Denmark, France, Germany, Italy, Poland, Slovenia, Spain, Sweden. The participants were instructed to wear a triaxial accelerometer (Axivity AX3, Axivity Ltd., Newcastle upon Tyne, United Kingdom) on their upper thigh for eight consecutive days, both day and night [19]. The Axivity AX3 is a valid instrument, and its reliability and validity have been confirmed in older adult populations [20,21]. The accelerometers were set to a sampling frequency of 50 Hz (with a range of ± 8 g). The raw accelerometer data were processed at SHARE central using ActiPASS Version 1.61beta, an open-source software based on the Acti4 algorithm for posture and activity recognition in data obtained from thigh-worn accelerometers [22]. The algorithms from ActiPASS were then used to identify 11 activities, including NonWear, Lie, Sit, Stand, Move, Walk, Run, Stair, Cycle, Other, Sleep, and LieStill. The time allocated to “LieStill”, “Sit” or “Lie” was considered as sedentary behavior (SEB). The time allocated to “Stand”, “Move”, “Walk Slow” (walking with a cadence lower than 100 steps/min), “Other” without any periodic movements, and “Other” with periodic movements at a cadence lower than 100 steps/min was classified as light physical activity (LPA). Moderate-to-vigorous physical activity (MVPA) was defined as the time allocated to “Run”, “Cycle”, “Stair”, “Walk” with a cadence above 100 steps/min, or “Other” activities with periodic movements at a cadence ≥100 steps/min. Additionally, sleep duration (SD) was determined by the time allocated to the “sleep” activity. Only participants with at least four days of data and 16 hours of wear each day were included in the analyses.

#### 2.2.2. Response variable.

In this study, eyesight, hearing, and self-perceived health were treated as dummy variables (categorical indicators). The participants’ distant and near vision were assessed utilizing two questions: “How would you rate your ability to see things at a distance, such as recognizing a friend across the street (with glasses or contact lenses if needed)?” and “How would you rate your ability to see things up close, such as reading regular newspaper text (with glasses or contact lenses if needed)?” The response options were “Excellent”, “Very good”, “Good”, “Fair”, and “Poor”. Similarly, hearing was evaluated by inquiring: “How would you rate your hearing (using a hearing aid if usual)?”. The options for this question were also “Excellent,” “Very good,” “Good,” “Fair,” and “Poor.”

The variable “Limitations with Activities” is a binary indicator, taking a value of 1 if the respondent reports being limited in performing activities typically done due to a health problem in the past six months and 0 otherwise. The scales were adapted from Katz S [23].

Self-perceived health refers to how individuals evaluate their health status. This variable was assessed by asking the question, “How would you rate your health?” The response options include “Excellent,” “Very good,” “Good,” “Fair,” and “Poor.”

### 2.3. Data analysis methods

The demographic data of the participants were presented as the mean ± standard deviation (M ± SD) for continuous variables and as percentages for categorical variables. The time allocated to each activity behavior was reported as a compositional mean. To explore the dependency structure among the four behaviors, we calculated a compositional variation matrix based on the variances of all pairwise log-ratios. Values close to 0 indicate that two behaviors tend to co-vary (i.e., they are more codependent), whereas larger values indicate greater relative dispersion between the two behaviors.

CoDA was applied to account for the relative and interdependent nature of 24-hour activity components, using log-ratio transformations to enable valid statistical inference. Linear compositional regression models (ordinary least squares) were built for each physical and sensory function. Linearity, independence, homoscedasticity, and normally distributed residuals assumptions were tested and showed satisfactory results. The time components for each activity were transformed into isometric log-ratio (ILR) coordinates and employed as response variables, with eyesight, hearing, limitations with activities, and self-perceived health as the predictors. The models were adjusted for the covariate variables total household net income, body mass index, age, sex, and country. The regression results are reported as coefficients [95% CI]. The adjusted estimated marginal means were computed and plotted for visual comparison. Marginal effects across each level were also reported. Type II MANOVA was used to test the overall association between each predictor and the 24-h activity composition. This step allowed us to determine whether, after adjustment, different levels of eyesight, hearing, functional limitation, or self-perceived health were globally related to how time was allocated across MVPA, LPA, SEB, and SD. The significance level was set at 0.05. P-values from pairwise post-hoc comparisons were adjusted using the Benjamini–Hochberg false discovery rate procedure to control for multiple testing. The R package “compositions” [24] was employed for the data analysis.

## 3. Results

### 3.1. Descriptive statistics

Table 1 provides an overview of the demographic characteristics, time spent on each activity, and physical health of the study participants. A total of 814 participants was included for analysis, comprising 41.0% males and 59.0% females, with an average age of 68.9 years. Participants originated from various countries: 15.0% from Poland, 13.5% from Germany, and only 4.4% from Denmark. Reported income showed substantial variability, with a large standard deviation relative to the mean, reflecting that a considerable proportion of participants reported zero income (e.g., not currently working or retired).

**Table 1:** Descriptive statistics results.

Variable name	Levels (N = 814)	Stats
**Demographic characteristics**		
Gender	Female	480 (59.0%)
	Male	334 (41.0%)
BMI, kg/m²	Mean ± SD	27.5 ± 4.9
Age, years	Mean ± SD	68.9 ± 8.9
Total household net income, EUR/year	Mean ± SD	25147.2 ± 24510.4
Number of chronic diseases	Mean ± SD	2.0 ± 1.5
Country	Belgium	76 (9.3%)
	Czech Republic	103 (12.7%)
	Denmark	36 (4.4%)
	France	75 (9.2%)
	Germany	110 (13.5%)
	Italy	66 (8.1%)
	Poland	122 (15.0%)
	Slovenia	97 (11.9%)
	Spain	66 (8.1%)
	Sweden	63 (7.7%)
**Sensory/Physical function variable**		
Eyesight	Poor	31 (3.8%)
	Fair	83 (10.2%)
	Good	301 (37.0%)
	Very good	235 (28.9%)
	Excellent	164 (20.1%)
Hearing	Poor	29 (3.6%)
	Fair	121 (14.9%)
	Good	327 (40.2%)
	Very good	210 (25.8%)
	Excellent	127 (15.6%)
Limitations with activities	Limited	408 (50.1%)
	Not limited	406 (49.9%)
Self-perceived health	Poor	60 (7.4%)
	Fair	211 (25.9%)
	Good	338 (41.5%)
	Very good	149 (18.3%)
	Excellent	56 (6.9%)

**Note:** BMI, Body mass index.

The majority of participants assessed their eyesight and hearing as “Good” or “Better”, with 700 participants (86%) reporting good or above eyesight and 664 participants (81.5%) reporting good or above hearing. Approximately half (408, 50.1%) reported experiencing some limitations in daily activities. Most participants evaluated their health as ranging from “Fair” to “Very good,” with only a small percentage rating their health as “Excellent” (56, 6.9%) or “Poor” (60, 7.4%).

Table 2 shown that descriptive statistics of 24-hour activity behaviors (minutes per day). Participants spent an average of 572.3 ± 125.0 minutes on SEB, which accounted for 43% of their daily time. This was followed by SD at 431.5 ± 99.0 minutes (32%) and LPA at 283.0 ± 110.8 minutes (21%). The time spent on MVPA was minimal, averaging 53.3 ± 30.6 minutes, representing only 4% of total time.

**Table 2:** Descriptive statistics of 24-hour activity behaviors (minutes per day).

Variable name	Levels (N = 814)	Stats
MVPA, mins/day	Mean ± SD	53.3 ± 30.6 (4%)
LPA, mins/day	Mean ± SD	283.0 ± 110.8 (21%)
SEB, mins/day	Mean ± SD	572.3 ± 125.0 (43%)
SD, mins/day	Mean ± SD	431.5 ± 99.0 (32%)

**Note:** MVPA, Moderate-to-vigorous physical activity; LPA, Light physical activity; SEB, Sedentary behavior; SD, Sleep duration.

### 3.2. Variance matrix

The variance matrix (Table 3) elucidates the pairwise log-ratio variances between activity behaviors. As anticipated, diagonal values were zero, indicating no intra-behavior variance. The off-diagonal values illustrate the extent of variability in the log ratios of time spent between pairs of behaviors.

**Table 3:** Compositional variation matrix of time spent in SEB, LPA, MVPA, and SD.

	SEB	LPA	MVPA	SD
SEB	0.00	0.40	1.02	0.16
LPA	0.40	0.00	0.64	0.37
MVPA	1.02	0.64	0.00	0.98
SD	0.16	0.37	0.98	0.00

**Note:** MVPA: Moderate-to-vigorous physical activity; LPA: Light physical activity; SEB: Sedentary behavior; SD: Sleep duration.

From the matrix, notably, MVPA consistently exhibits the highest log-ratio variances with other behaviors (e.g., SEB: 1.02, SD: 0.98), suggesting it is the least codependent with other activities. In contrast, lower variances observed between LPA and SEB (0.40) or SD (0.37) imply a stronger codependent effect, indicating that changes in one of these behaviors are more likely to correspond to proportional adjustments in the other.

### 3.3. Compositional data analysis results

**Fig 1:**
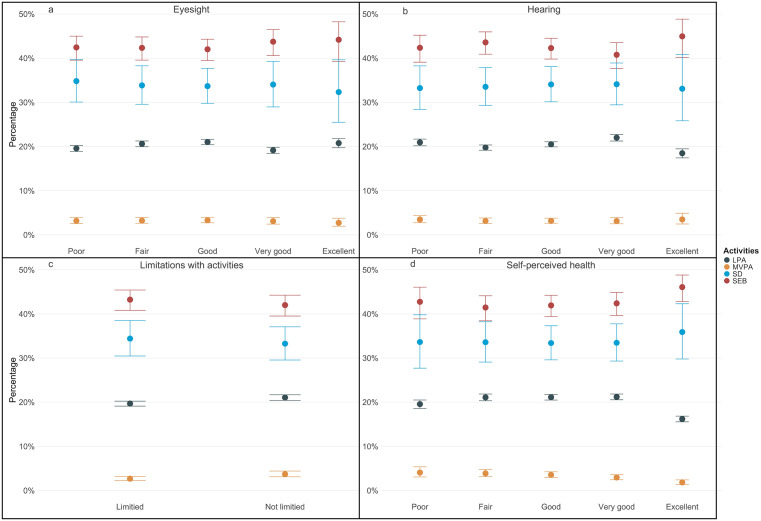
Predictions and 95% CIs of the four activities for eyesight, hearing, limitation with activities, and self-perceived health.

Table 1 of S1 Appendix presents regression model results. Type II MANOVA results indicated that limitations with activities and self-perceived health is significantly associated with 24h activity behaviors but hearing and eyesight is not significantly associated with 24h activity behaviors (Table 2 in S1 Appendix). Fig 1 presents the proportion of time that older adults with different levels of sensory and physical function allocated to various activity behaviors. Prediction values can be found in Table 3 of S1 Appendix. Fig 1a shows group differences in activity allocation across eyesight levels. Participants reporting excellent eyesight spent similar proportions of time in MVPA, LPA, SEB, and SD compared with those reporting poor eyesight, with no statistically significant differences observed (Table 4). Hearing showed a similar pattern (Fig 1b; Table 4).

**Table 4:** Marginal effects and 95% CIs for MVPA, LPA, SEB, and SD across eyesight, hearing, limitations with activities, and self-perceived health.

	MVPA			LPA			SEB			SD		
Contrast	Estimate	95%CI	p.value	Estimate	95%CI	p.value	Estimate	95%CI	p.value	Estimate	95%CI	p.value
**Self-Perceived Health**												
Fair – Poor	0.38	0.18, 0.58	0.001	0.14	0.03, 0.25	0.065	−0.27	−0.39, −0.14	0.000	−0.25	−0.38, −0.13	0.000
Good – Poor	0.54	0.34, 0.75	0.000	0.09	−0.03, 0.20	0.555	−0.33	−0.46, −0.20	0.000	−0.30	−0.43, −0.18	0.000
Very good – Poor	0.62	0.39, 0.85	0.000	0.06	−0.07, 0.19	1.000	−0.36	−0.51, −0.22	0.000	−0.32	−0.46, −0.18	0.000
Excellent – Poor	0.68	0.40, 0.96	0.000	−0.03	−0.18, 0.13	1.000	−0.33	−0.50, −0.16	0.001	−0.32	−0.49, −0.15	0.001
**Eyesight**												
Fair – Poor	0.12	−0.17, 0.42	1.000	−0.12	−0.28, 0.04	0.610	−0.04	−0.22, 0.15	1.000	0.03	−0.14, 0.21	1.000
Good – Poor	0.16	−0.10, 0.43	0.910	−0.04	−0.19, 0.10	1.000	−0.11	−0.28, 0.05	0.704	−0.01	−0.17, 0.15	1.000
Very good – Poor	0.14	−0.13, 0.41	1.000	−0.06	−0.20, 0.09	1.000	−0.09	−0.26, 0.07	1.000	0.01	−0.16, 0.17	1.000
Excellent – Poor	0.14	−0.14, 0.42	1.000	−0.11	−0.26, 0.05	0.722	−0.08	−0.26, 0.09	1.000	0.05	−0.12, 0.22	1.000
**Hearing**												
Fair – Poor	−0.12	−0.42, 0.17	1.000	0.20	0.04, 0.36	0.055	−0.11	−0.29, 0.07	0.886	0.03	−0.14, 0.21	1.000
Good – Poor	−0.11	−0.38, 0.17	1.000	0.13	−0.02, 0.28	0.381	−0.06	−0.23, 0.11	1.000	0.04	−0.13, 0.21	1.000
Very good – Poor	−0.11	−0.39, 0.18	1.000	0.09	−0.06, 0.25	0.956	−0.02	−0.19, 0.16	1.000	0.03	−0.14, 0.20	1.000
Excellent – Poor	−0.02	−0.32, 0.28	1.000	0.13	−0.04, 0.29	0.540	−0.09	−0.27, 0.10	1.000	−0.01	−0.20, 0.17	1.000
**Limitations with activities**												
Not limited – Limited	0.20	0.09, 0.32	0.001	0.00	0.07, 0.06	0.903	−0.09	−0.17	0.019	−0.11	−0.23	0.003

**Note:** MVPA, Moderate-to-vigorous physical activity; LPA, Light physical activity; SEB, Sedentary behavior; SD, Sleep duration.

As shown in Fig 1c, compared to participants with activity limitations, participants without activity limitations allocate more time to MVPA (P-value = 0.001) and less time on SEB (P-value = 0.019) and SD (P-value = 0.003) (Table 4).

Finally, Fig 1d shows that self-perceived health is positively correlated with time spent in MVPA. Compared with participants with “poor” self-perceived health, participants with other self-perceived health spent significantly more time in MVPA (P-value < 0.001), and less time on SEB (P-value < 0.001) and SD (P-value < 0.001) (Table 4). However, self-perceived health was not significantly correlated with time spent in LPA (P-value range from 0.065 to 1.000) (Table 4).

## 4. Discussion

This study, utilizing the SHARE database, examined 24-hour activity behaviors across different levels of sensory/physical function through CoDA methodologies. The results suggest that variations in vision and hearing status are unlikely to significantly associated with the daily time distribution of older adults. Conversely, self-perceived health and activity limitations substantially associated with time allocation of time across various activity behaviors. Specifically, enhanced self-perceived health and reduced activity limitations are associated with more time dedicated to MVPA and LPA, while decreasing time spent on SD and SEB.

Prior studies have linked vision impairment or decline to LPA, SEB, and SD [25–27]. It has been shown that individuals with vision impairments are twice as likely to be inactive compared to those with normal vision [8]. Furthermore, people with self-reported “fair-poor” eyesight, even while wearing glasses or contact lenses, are similarly more prone to inactivity than those who rated their eyesight as “excellent” [26]. Other studies have also highlighted the association between vision impairment and SD [28]. Likewise, hearing loss has been found to be strongly related to older adults’ lifestyle behaviors, particularly with higher levels of SEB [7,29,30]. Additionally, lower hearing ability is significantly linked to reduced physical activity [30]. However, the results of this study reveal that self-reported vision and hearing status are not associated with daily time allocated to activity behaviors among older adults. One possible explanation is that the SHARE database assesses vision and hearing not by objective sensory tests (e.g., visual acuity charts or audiometry). In other words, these measures emphasize functional performance rather than precise sensory acuity. As a result, the vision and hearing variables employed here may not accurately reflect true sensory capacity. Another possible explanation is that adults’ activity behaviors are related to a wider range of complex factors, with vision and hearing potentially being only one of many. In fact, older adults’ activity behaviors may be more constrained by a general decline in sensory/physical function, such as joint issues, cardiovascular deterioration, and muscle weakness. Additionally, reductions in PA among older adults may be more closely tied to psychological and social factors, such as fear of falling, lack of social support, and limited opportunities for exercise, which may have a greater impact on activity behaviors than vision and hearing status. Thus, even though vision impairment may be associated with PA to some extent, its effects in older populations may be overshadowed by other, more dominating limiting factors. Consistent with the previous hypothesis, the study has revealed that limitations are strongly associated with PA behaviors, aligning with previous research [31].

Previous studies have shown a positive correlation between self-perceived health and PA and SD in older adults [32], while demonstrating a negative correlation with SEB [33–36]. These findings align with the results of this investigation. However, for those who rated their health as “fair” to “very good,” there was no notable difference in time allocation. This indicates that the relationship between self-perceived health and activity behaviors may not be linear. One reason for this is that, despite some functional limitations, these constraints may not yet be substantial enough to severely be related to their daily activity level. Simultaneously, they are probably aware of the potential health risks associated with their condition. These individuals may proactively adjust their activity behaviors, for instance, by avoiding excessive high-intensity activities to reduce physical strain while maintaining their health through moderate daily activities, such as walking or household chores. This “balancing strategy” may lead to a more even and similar distribution of activity time.

An alternative explanation is that older adults with intermediate self–rated health may lack sufficient motivation to substantially increase their activity levels. Compared with those who rate their health as “Excellent,” they may have reduced confidence or perceive greater physical limitations, yet their functional status is not so compromised as to require external assistance—as is often the case for individuals who rate their health as “Poor.” As a result, their habitual activity patterns remain relatively stable. Additionally, because our sample was drawn from high-income European countries—where older adults typically benefit from stable social support and accessible activity environments [37,38] —these resources likely help maintain existing activity levels despite minor health concerns, without necessarily promoting higher-intensity or more frequent physical activity.

Interestingly, the older adults with the highest self-perceived health scores presented lower levels of LPA and greater SEB. This may indicate an overestimation of their physical condition, resulting in the belief that additional activity is unnecessary for health maintenance, thereby favoring sedentary activities such as socializing, reading, or leisure activities. Furthermore, they may have fulfilled their daily activity requirements through extended durations of SD and moderate-to-high intensity exercise, leading to a reduction in low-intensity activities. However, these interpretations remain speculative, and there is currently no direct evidence to support their validity. Future research needs to further explore this issue. Longitudinal studies are required to investigate the causal relationship between self-perceived health and specific activity behavior patterns, clarifying whether high self-perceived health is indeed associated with activity behaviors. Additionally, qualitative research (e.g., interviews or surveys) may elucidate the motivational factors and lifestyle preferences of individuals with high self-perceived health, thereby shedding light on the potential drivers of their activity behaviors.

### 4.1. Implications

This study has several important implications. Initially, we discovered that participants with varying degrees of vision and hearing may not display markedly different activity behavior patterns. Second, limitations in activities play a crucial role in shaping the activity patterns of older adults. Future interventions should specifically target older adults with activity limitations through health‐promotion initiatives that encourage physical activity in accordance with established guidelines. Finally, it was noted that, apart from those with the poorest self-rated health, older adults across diverse self-perceived health levels tend to exhibit comparable activity behavior patterns. This suggests that adaptive behavior adjustments, a lack of motivation, and the balance of social support may jointly contribute to the stability of their activity time allocation.

### 4.2. Strengths

This study is the first to examine the relationships between various sensory and physical functions in older adults and their 24-hour movement behaviors. Unlike previous studies that treated PA, SEB, and SD as independent variables, this study employs a CoDA framework to acknowledge the interdependent nature of these behaviors throughout a 24-hour period. This innovative methodology addresses prevalent statistical issues, including collinearity, and provides a more holistic understanding of how functions shape overall lifestyle patterns. Furthermore, by identifying specific time allocation patterns among older adults with varying function levels, this study offers valuable insights for developing more tailored and actionable health guidelines. These findings may inform targeted interventions, mitigate challenges faced by older adults, and promote healthier lifestyles within this population.

### 4.3. Limitations

First, the cross-sectional design precludes definitive causal inference between 24-hour activity behaviors and sensory/physical function in older adults, and it is plausible that participants’ baseline health status drove their self-reported activity levels. Secondly, the study has a rather limited sample size. This constraint precluded the investigation of potential moderating factors. Subsequent research should utilize larger sample sizes and explore the impact of sensory/physical function on older adults’ activity behaviors across different demographics, such as gender, age, BMI, and nation. Moreover, one limitation of this study is that the “number of chronic diseases” variable does not differentiate between disease types or severities, which may limit the precision of this measure. In addition, distant and near vision and hearing in this study were assessed via highly subjective, self-reported measures rather than objective clinical tests. Lastly, potential confounding variables that could influence both sensory/physical function and self-perceived health—such as comorbidities, medication use, socioeconomic status, and lifestyle factors—were not fully accounted for. Moreover, employing a relatively straightforward linear regression model limits the ability to comprehensively capture the complexity of the data. Finally, the regional characteristics of the sample restrict the generalizability of the findings to a broader population, encompassing individuals beyond Europe and other areas.

## 5. Conclusions

This study revealed that vision and hearing conditions may not significantly affect health activities in older adults. Nonetheless, self-perceived health, limitations in daily activities, and restrictions in activities have a big influence on their daily activity patterns. Specifically, greater self-perceived health and fewer activity limitations are associated with increased duration of MVPA and LPA, alongside diminished time spent on SD and SEB. The findings provide novel insights into the relationship between sensory/physical function and 24-hour movement behavior in older adults. Future research should consider longitudinal studies to further explore the causal relationship between self-perceived health and specific activity behavior patterns, while also investigating potential moderating factors, such as age and BMI.

## Supporting information

S1 Appendix **Table 1.** Regression coefficients (ilr) and 95% confidence intervals: associations between sociodemographic and health factors and MVPA, LPA, SEB, and SD. **Table 2.** Multivariate F test results. **Table 3.** Regression estimates (adjusted marginal mean) and 95% CIs of time spent in MVPA, LPA, SEB, and SD across different levels of eye sight, hearing, limitations with activities, and self-perceived health.
(DOCX)

## Abbreviations

BMI: Body mass index
ILR: Isometric log ratio
MVPA: Moderate-to-vigorous physical activity
LPA: Light physical activity
SEB: Sedentary behavior
SD: Sleep duration
CoDA: Compositional Data Analysis
SHARE: Survey of Health, Aging and Retirement in Europe
