# Supplementary material for: The association between physical and sensory function, self-perceived health, and 24-hour activity patterns for older people: A compositional data analysis
Source: PLoS One. 2025 Dec 31;20(12):e0340216. doi: 10.1371/journal.pone.0340216 (PMC12755779; doi:10.1371/journal.pone.0340216)
Supplement: S1 Appendix — Table 1. Regression coefficients (ilr) and 95% confidence intervals: associations between sociodemographic and health factors and MVPA, LPA, SEB, and SD. Table 2. Multivariate F test results. Table 3. Regression estimates (adjusted marginal mean) and 95% CIs of time spent in MVPA, LPA, SEB, and SD across different levels of eye sight, hearing, limitations with activities, and self-perceived health. (DOCX) [file pone.0340216.s001.docx]

**Appendix**

**Table 1 Regression coefficients (ilr) and 95% confidence intervals: associations between sociodemographic and health factors and MVPA, LPA, SEB, and SD**

| **Variables** | **Level** | **MVPA** | | | **LPA** | | | **SEB** | | | **SD** | | |
| --- | --- | --- | --- | --- | --- | --- | --- | --- | --- | --- | --- | --- | --- |
|  |  | **Coefficient (ilr1)** | **95%CI** | **P value** | **Coefficient (ilr1)** | **95%CI** | **P value** | **Coefficient (ilr1)** | **95%CI** | **P value** | **Coefficient (ilr1)** | **95%CI** | **P value** |
| Limitation with activities | Reference (Limited) | | | | | | | | | | | | |
|  | Not limited | 0.20 | 0.09, 0.32 | 0.004 | 0.00 | -0.07, 0.06 | 0.973 | -0.09 | -0.16, -0.01 | 0.068 | -0.11 | -0.19, -0.04 | 0.015 |
| Self-perceived health | Reference (Poor) | |  |  |  |  |  |  |  |  |  |  |  |
|  | Fair | 0.37 | 0.16, 0.57 | 0.003 | 0.14 | 0.02, 0.25 | 0.103 | -0.25 | -0.38, -0.12 | <0.001 | -0.25 | -0.38, -0.13 | 0.001 |
|  | Good | 0.46 | 0.25, 0.68 | <0.001 | 0.09 | -0.03, 0.21 | 0.361 | -0.29 | -0.42, -0.15 | <0.001 | -0.26 | -0.39, -0.13 | 0.001 |
|  | Very good | 0.50 | 0.25, 0.75 | 0.001 | 0.07 | -0.07, 0.20 | 0.525 | -0.31 | -0.47, -0.16 | <0.001 | -0.25 | -0.41, -0.10 | 0.007 |
|  | Excellent | 0.53 | 0.23, 0.83 | 0.003 | -0.02 | -0.18, 0.15 | 0.951 | -0.27 | -0.45, -0.08 | 0.017 | -0.25 | -0.43, -0.06 | 0.036 |
| Eyesight | Reference (Poor) | | | | | | | | | | | | |
|  | Fair | 0.11 | -0.18, 0.40 | 0.681 | -0.12 | -0.29, 0.04 | 0.344 | -0.02 | -0.20, 0.16 | 0.858 | 0.04 | -0.14, 0.22 | 0.774 |
|  | Good | 0.09 | -0.18, 0.35 | 0.694 | -0.04 | -0.19, 0.10 | 0.716 | -0.07 | -0.23, 0.09 | 0.716 | 0.03 | -0.13, 0.19 | 0.774 |
|  | Very good | 0.05 | -0.22, 0.32 | 0.840 | -0.05 | -0.20, 0.11 | 0.716 | -0.06 | -0.23, 0.11 | 0.776 | 0.05 | -0.11, 0.22 | 0.710 |
|  | Excellent | 0.02 | -0.25, 0.30 | 0.902 | -0.10 | -0.25, 0.06 | 0.442 | -0.04 | -0.21, 0.14 | 0.858 | 0.11 | -0.06, 0.28 | 0.473 |
| Hearing | Reference (Poor) | | | | | | | | | | | | |
|  | Fair | -0.18 | -0.46, 0.11 | 0.541 | 0.19 | 0.03, 0.35 | 0.103 | -0.08 | -0.26, 0.10 | 0.716 | 0.07 | -0.11, 0.24 | 0.710 |
|  | Good | -0.15 | -0.42, 0.12 | 0.610 | 0.12 | -0.03, 0.27 | 0.344 | -0.04 | -0.21, 0.13 | 0.858 | 0.07 | -0.10, 0.23 | 0.710 |
|  | Very good | -0.18 | -0.47, 0.10 | 0.541 | 0.09 | -0.07, 0.25 | 0.476 | 0.03 | -0.15, 0.21 | 0.858 | 0.06 | -0.11, 0.24 | 0.710 |
|  | Excellent | -0.09 | -0.39, 0.21 | 0.711 | 0.13 | -0.03, 0.30 | 0.344 | -0.05 | -0.23, 0.14 | 0.858 | 0.00 | -0.18, 0.18 | 0.977 |
| Gender | Reference (Female) | | | | | | | | | | | | |
|  | Male | 0.04 | -0.06, 0.14 | 0.640 | -0.17 | -0.22, -0.11 | <0.001 | 0.15 | 0.09, 0.21 | <0.001 | -0.03 | -0.09, 0.03 | 0.710 |
| Age | Age | -0.03 | -0.04, -0.03 | <0.001 | 0.00 | 0.00, 0.01 | 0.175 | 0.01 | 0.01, 0.02 | <0.001 | 0.02 | 0.01, 0.02 | <0.001 |
| Country | Reference (Belgium) | | | | | | | | | | | | |
|  | Czech Republic | 0.04 | -0.18, 0.26 | 0.840 | 0.07 | -0.05, 0.20 | 0.473 | -0.01 | -0.15, 0.13 | 0.929 | -0.11 | -0.24, 0.03 | 0.361 |
|  | Denmark | 0.04 | -0.24, 0.32 | 0.865 | -0.15 | -0.31, 0.01 | 0.205 | 0.16 | -0.01, 0.34 | 0.161 | -0.05 | -0.23, 0.12 | 0.710 |
|  | France | 0.19 | -0.03, 0.41 | 0.297 | 0.00 | -0.13, 0.13 | 0.994 | -0.14 | -0.28, 0.00 | 0.133 | -0.05 | -0.19, 0.09 | 0.710 |
|  | Germany | 0.16 | -0.05, 0.36 | 0.393 | 0.00 | -0.12, 0.12 | 0.994 | -0.05 | -0.18, 0.07 | 0.716 | -0.10 | -0.23, 0.02 | 0.361 |
|  | Italy | 0.00 | -0.24, 0.24 | 0.994 | 0.27 | 0.14, 0.41 | 0.001 | -0.17 | -0.32, -0.02 | 0.087 | -0.11 | -0.25, 0.04 | 0.394 |
|  | Poland | 0.09 | -0.13, 0.31 | 0.640 | -0.02 | -0.14, 0.11 | 0.935 | -0.03 | -0.17, 0.11 | 0.858 | -0.05 | -0.18, 0.09 | 0.710 |
|  | Slovenia | 0.11 | -0.12, 0.33 | 0.618 | 0.08 | -0.04, 0.21 | 0.404 | -0.07 | -0.21, 0.07 | 0.657 | -0.12 | -0.26, 0.02 | 0.342 |
|  | Spain | 0.11 | -0.13, 0.36 | 0.618 | -0.07 | -0.20, 0.07 | 0.517 | -0.08 | -0.23, 0.07 | 0.652 | 0.04 | -0.11, 0.18 | 0.774 |
|  | Sweden | 0.21 | -0.03, 0.45 | 0.297 | -0.07 | -0.20, 0.06 | 0.495 | -0.02 | -0.17, 0.13 | 0.858 | -0.11 | -0.26, 0.03 | 0.361 |
| BMI | BMI | -0.01 | -0.02, 0.00 | 0.297 | -0.01 | -0.02, 0.00 | 0.003 | 0.02 | 0.01, 0.03 | <0.001 | 0.00 | -0.01, 0.01 | 0.881 |
| Total household net income | Total household net income | 0.00 | 0.00, 0.00 | 0.694 | 0.00 | 0.00, 0.00 | 0.935 | 0.00 | 0.00, 0.00 | 0.858 | 0.00 | 0.00, 0.00 | 0.710 |
| Number of chronic diseases | Number of chronic diseases | 0.02 | -0.02, 0.06 | 0.618 | -0.03 | -0.05, -0.01 | 0.025 | 0.01 | -0.02, 0.03 | 0.858 | 0.01 | -0.02, 0.03 | 0.770 |

**Note:** MVPA, Moderate-to-vigorous physical activity; LPA, Light phys-ical activity; SEB, Sedentary behavior; SD, Sleep duration.

**Table 2 Multivariate F test results**

| Variables | F | Pr(>F) |
| --- | --- | --- |
| Limitations with activities | 4.01 | 0.008 |
| Self-perceived health | 2.65 | 0.002 |
| Eyesight | 0.83 | 0.625 |
| Hearing | 1.43 | 0.146 |
| Gender | 21.11 | <0.001 |
| Age | 37.14 | <0.001 |
| Country | 2.75 | <0.001 |
| BMI | 16.31 | <0.001 |
| Total household net income | 0.29 | 0.830 |
| Number of chronic disease | 2.93 | 0.033 |

**Table 3 Regression estimates (adjusted marginal mean) and 95% CIs of time spent in MVPA, LPA, SEB, and SD across different levels of eye sight, hearing, limitations with activities, and self-perceived health**

| **Level** | **MVPA** | **95%CI** | **LPA** | **95%CI** | **SEB** | **95%CI** | **SD** | **95%CI** |
| --- | --- | --- | --- | --- | --- | --- | --- | --- |
| **Eyesight** | | | | | | | | |
| Poor | 2.83% | 1.99%, 3.98% | 21.25% | 17.98%, 24.71% | 43.23% | 42.34%, 43.43% | 32.68% | 37.70%, 27.88% |
| Fair | 3.17% | 2.42%, 4.11% | 19.40% | 17.07%, 21.85% | 43.06% | 42.31%, 43.41% | 34.37% | 38.20%, 30.63% |
| Good | 3.13% | 2.50%, 3.89% | 20.95% | 18.85%, 23.13% | 41.60% | 41.07%, 41.85% | 34.32% | 37.59%, 31.13% |
| Very good | 3.01% | 2.35%, 3.83% | 20.71% | 18.41%, 23.11% | 41.57% | 40.93%, 41.87% | 34.71% | 38.31%, 31.19% |
| Excellent | 2.88% | 2.22%, 3.72% | 19.48% | 17.17%, 21.91% | 41.79% | 40.99%, 42.23% | 35.85% | 39.63%, 32.14% |
| **Hearing** | | | | | | | | |
| Poor | 3.64% | 2.51%, 5.18% | 19.35% | 16.22%, 22.65% | 43.90% | 42.89%, 44.11% | 33.12% | 38.38%, 28.06% |
| Fair | 3.06% | 2.35%, 3.96% | 22.34% | 19.71%, 25.08% | 40.10% | 39.50%, 40.30% | 34.49% | 38.44%, 30.65% |
| Good | 3.13% | 2.50%, 3.89% | 20.95% | 18.85%, 23.13% | 41.60% | 41.07%, 41.85% | 34.32% | 37.59%, 31.13% |
| Very good | 2.99% | 2.33%, 3.80% | 20.11% | 17.88%, 22.43% | 43.26% | 42.62%, 43.55% | 33.65% | 37.17%, 30.21% |
| Excellent | 3.35% | 2.53%, 4.39% | 21.66% | 19.00%, 24.42% | 41.92% | 41.33%, 42.06% | 33.07% | 37.13%, 29.13% |
| **Limitations with activities** | | | | | | | | |
| Limited | 3.13% | 2.50%, 3.89% | 20.95% | 18.85%, 23.13% | 41.60% | 41.07%, 41.85% | 34.32% | 37.59%, 31.13% |
| Not limited | 3.96% | 3.23%, 4.83% | 22.14% | 20.15%, 24.18% | 40.86% | 40.54%, 40.93% | 33.04% | 36.09%, 30.06% |
| **Self-Perceived Health** | | | | | | | | |
| Poor | 1.78% | 1.32%, 2.38% | 16.47% | 14.22%, 18.91% | 45.22% | 43.94%, 46.10% | 36.53% | 40.52%, 32.62% |
| Fair | 2.82% | 2.25%, 3.50% | 21.36% | 19.23%, 23.58% | 41.92% | 41.39%, 42.17% | 33.90% | 37.13%, 30.75% |
| Good | 3.13% | 2.50%, 3.89% | 20.95% | 18.85%, 23.13% | 41.60% | 41.07%, 41.85% | 34.32% | 37.59%, 31.13% |
| Very good | 3.27% | 2.49%, 4.26% | 20.76% | 18.23%, 23.39% | 41.07% | 40.38%, 41.36% | 34.90% | 38.90%, 31.00% |
| Excellent | 3.34% | 2.37%, 4.62% | 19.24% | 16.35%, 22.30% | 42.45% | 41.43%, 42.84% | 34.97% | 39.84%, 30.24% |

**Note:** MVPA, Moderate-to-vigorous physical activity; LPA, Light phys-ical activity; SEB, Sedentary behavior; SD, Sleep duration.
